# Als3‐Th‐cell‐epitopes plus the novel combined adjuvants of CpG, MDP, and FIA synergistically enhanced the immune response of recombinant TRAP derived from *Staphylococcus aureus* in mice

**DOI:** 10.1002/iid3.456

**Published:** 2021-05-19

**Authors:** Jinzhu Ma, Wei Liu, Beiyan Wang, Simiao Yu, Liquan Yu, Baifen Song, Yongzhong Yu, Zhanbo Zhu, Yudong Cui

**Affiliations:** ^1^ College of Life Science and Technology Bayi Agricultural University Daqing Heilongjiang China; ^2^ College of Animal Science and Veterinary Medicine Bayi Agricultural University Daqing Heilongjiang China

**Keywords:** Als3 epitopes, ATT, Combined adjuvants, CpG, FIA, MDP

## Abstract

**Introduction:**

*Staphylococcus aureus* (*S. aureus*) is a gram‐positive opportunistic pathogen, there are currently no high effective vaccine against *S. aureus* in humans and animals, the development of an efficient vaccine remains an important challenge to prevent *S. aureus* infection. Here, we prepared Als3‐Th‐cell‐epitope‐Target of RNAIII Activating Protein (TRAP) (ATT) proteins plus the novel combined adjuvants to develop a promising vaccine candidate against *S. aureus*.

**Methods:**

The recombinant pET‐28a (+)‐*att* plasmids were constructed, and the ATT proteins were expressed and obtained, then, ATT plus Freund's adjuvant or the novel combined adjuvants of cytosine‐phosphate‐guanosine oligodeoxynucleotides (CpG), muramyl dipeptides (MDP), and FIA were immunized in mice. After booster immunization, the levels of interferon‐γ (IFN‐γ), interleukin‐4 (IL‐4), IL‐10 and IL‐17A cytokine were evaluated, the humoral immune responses against TRAP were detected in mice, and the survival rate of mice was confirmed by challenge assay.

**Results:**

The mice immunized with ATT plus Freund's adjuvant exhibited significantly higher level of IFN‐γ, IL‐4, IL‐10, and IL‐17A, and displayed the stronger humoral immune response against TRAP than control groups, importantly, the survival rate of these mice was significantly higher than control groups. In addition, compared with the control groups, ATT + CpG + MDP + FIA group was elicited significantly higher level of IFN‐γ, IL‐4, IL‐10, and IL‐17A and was triggered the stronger humoral immune responses against TRAP, moreover, generated the higher survival rate of mice.

**Conclusion:**

Als3 epitopes significantly enhanced TRAP immunogenicity. ATT plus the novel combined adjuvants of CpG, MDP, and FIA induced the strong immune response and protection against *S. aureus*, revealing the combination of CpG, MDP, and FIA adjuvant acts the synergistic effect.

## Graphical abstract

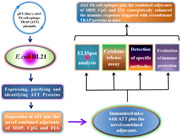

## 1 INTRODUCTION

*Staphylococcus aureus* (*S. aureus*) is an important opportunistic pathogen, which is distributed in water, dust, and other natural environments, and also exists in human and animal skin, excreta, and cavities.^1^, ^2^, ^3^ *S. aureus* commonly causes the pneumonia, endocarditis, osteomyelitis and other diseases in human,^4^, ^5^, ^6^, ^7^ it also elicits mastitis in sheep and bovine, and canine pyoderma.^8^, ^9^, ^10^ Over the years, the increasing emergence of resistant strains, such as methicillin‐resistant *Staphylococcus aureus* (MRSA) and vancomycin‐resistant *S. aureus*, was attributed to the excessive use of antibiotics in many countries and regions.^11^, ^12^, ^13^, ^14^ Clinical studies have shown that the vaccine is effective in preventing *S. aureus* infection, therefore, developing an effective vaccine against *S. aureus* is urgently needed.^15^, ^16^, ^17^

Target of RNAIII Activating Protein (TRAP) of *S. aureus* is a membrane‐bound protein of 167 amino acid residues, which is relatively conservative and is consistently expressed. TRAP activates downstream proteins by binding RAP, which in turn can activate and promote the synthesis of RNAIII, and ultimately increase the toxin expression. Kiran and Balaban^18^ have shown that TRAP can protect DNA from natural mutations, adaptive mutations, and oxidative damage during the stress response of *S. aureus*. The mice immunized with TA21 peptide from TRAP generated immune protective response against *S. aureus* infection, and TRAP triggered the strong immune protection and the high level of IFN‐γ, IL‐4, and IL‐17^19^ in mice. Therefore, TRAP displays the strong immunogenicity, however, its immunogenicity will still needed to be further increased to prevent effectively *S. aureus* infection.

Als3 (Agglutinin‐like sequence 3), a critical adhesion factor, plays a crucial role of improving *Candida albicans* (*C. albicans*) to adhere the surfaces of host cells,^20^, ^21^, ^22^ and its three‐dimensional structures are similar to clumping factor A (ClfA) of *S. aureus*.^23^ Preclinical studies demonstrated that Als3p can protect mice from intravenous challenge with *C. albicans* and *S. aureus*, and promote the secretion of interferon‐γ (IFN‐γ) and IL‐17A,^20^, ^22^, ^24^ indicating Als3p elicits the immune cross‐reaction against *S. aureus* and *Candida* infection. Additionally, Bar et al.^25^ found that an Als3‐Th‐cell‐epitopes (Als3 epitopes) from Als3 proteins induced the immune protective response against *C. albicans*.

Unmethylated cytosine‐phosphate‐guanosine oligodeoxynucleotides (CpG‐ ODN (CpG)), as agonists of Toll‐like receptor 9 (TLR9), activate B cells and dendritic cells, and induce Th1‐mediated immune response.^26^, ^27^, ^28^, ^29^, ^30^ In addition, muramyl dipeptides (MDP) promotes the secretion of proinflammatory cytokines through binding NOD2 receptor, then, improves cellular immune response, mainly inducing Th1‐mediated cellular immune response.^31^, ^32^, ^33^, ^34^ Freund's incomplete adjuvant (FIA) is an oil–water–emulsion emulsified with oil (paraffin oil or vegetable oil) and emulsifier (lanolin or Twin‐80), as commonly used adjuvant in animal experiments. FIA sustains slow release of antigen and enhances the immunogenicity of antigens by upregulating Th2‐mediated immune response.^35^, ^36^ However, an adjuvant often triggers a weak immune response against antigens, the combined adjuvants act synergistic effect to maximize the immunogenicity of antigens. Hence, we predicted that the combination of CPG, MDP, and FIA adjuvant could generate a synergistic function to enlarge immune response against antigens, which would be responsible for driving the polarization of naïve CD_4_^+^ T cells toward Th1, Th2, Th17 cells.

In this study, Als3‐Th‐cell‐epitope‐TRAP (ATT) fusion proteins were expressed and prepared, then, Als3 epitopes in combination with CPG, MDP, and FIA adjuvant acted synergistic effects to enlarge the immune response against TRAP.

## 2 MATERIALS AND METHODS

### 2.1 Mice and bacterial strains

Female C57/B6 mice (6–8 weeks of age) were ordered from the Changchun Institute of Biological Products. Animal experiment was performed in accordance with animal ethics guidelines approved by the Animal Ethics Committee of Heilongjiang Bayi Agricultural University. *S. aureus* strain Newman and *S. aureus* strain Wood46 were grown in tryptic soy agar (TSA), and *Escherichia coli* strain BL21 was grown in Luria‐Bertani (LB) broth at 37°C overnight.

### 2.2 Construction of recombinant plasmids

**Figure 1:**
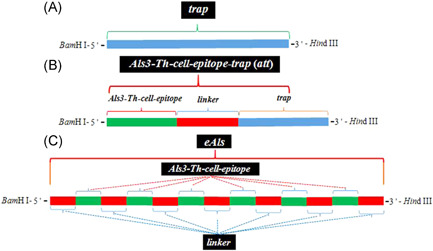
The structural cassette of target gene. The target gene carries *Bam* H I, *Hin*d III restriction endonuclease at 5′ terminal and 3′ terminal, respectively. (A) The cassette of*trap*gene. The *trap* gene involves in 498 bp. (B) The cassette of*att*gene. The *att* gene of 558 bp includes three parts, *Als3‐Th‐cell‐epitope*, *linker*, and *trap*. (C) The cassette of*eAls*gene. The *eAls* gene consists of 426 bp, containing *Als3‐Th‐cell‐epitope* repeated for six times and the nucleotide sequence of flexible linker repeated for seven times

The *trap* genes were obtained by polymerase chain reaction (PCR) with forward primer 5′‐ GGATCCAAGAAACTATATACATCTT‐3′ (*Bam*H I site underlined) and reverse primer AAGCTTTTCTTTTATTGGGTAT (*Hin*d III site underlined) from the pET‐32a (+)‐*trap* plasmids, the structural cassette of *trap* gene was shown in Figure 1A. The PCR conditions were as follows: denaturation at 94°C for 5 min; followed by 30 cycles of 94°C, 45 s; 56°C, 40 s; 72°C, 40 s; extension at 72°C for 8 min. Finally, the *trap* fragments were linked into pET‐28a (+) vectors.

The *Als3‐Th‐cell‐epitope* (*Als3‐epitope*)*‐trap* (*att*) genes were acquired from the pET‐28a (+)‐*trap* plasmid by PCR with forward primer 5′‐ *GGATCCTGGAATTATC‐*

*CGGTTTCATCTGAATCA* GGTAGTGGTAGTGGTAGTGGTAGTAAGAAACTATATACATCTT‐3′ (*Bam*H I site with italicized and underlined, *Als3‐epitope* sequence with italicized, linker sequence underlined) and reverse primer 5′‐ AAGCTTTTCTTTTATTGGGTAT‐3′ (*Hin*d III site underlined), the structural cassette of *att* was exhibited in Figure 1B. The PCR conditions were as follows: denaturation at 94°C for 5 min; followed by 30 cycles of 94°C, 45 s; 58°C, 40 s; 72°C, 40 s; extension at 72°C for 8 min. The *att* fragments were inserted into pET‐28a (+) vectors.

*eAls* (epitope *Als*) gene includes the DNA sequence that *Als3‐epitope* (TGGAATTATCCGGTTTCATCTGAATCA) connected with flexible linker (GGTGGTAGCGGTGGCGGTTCTGGTGGCGGCTCTGGT) was repeated for six times, and the same linker was added to the 5′‐terminal of the first *Als3‐epitope* and the 3′‐terminal of the last *Als3‐epitope*, and the sites of *Bam*H I and *Hin*d III restriction endonucleases were added at 5′‐terminal and 3′‐terminal of the whole sequence, respectively, and the structural cassette of *eAls* gene was shown in Figure 1C. *Als* genes were synthesized by Sangon Biological Engineering Technology Service Co., LTD, and inserted into pET‐28a (+) plasmids.

### 2.3 Expression, purification, and analysis of proteins

The recombinant plasmids, pET‐28a (+)‐*att*, pET‐28a (+)‐*trap*, and pET‐28a (+)‐*eAls*, were transformed into in *Escherichia coli* BL21 (DE3) (Tiangen) and were expressed the ATT, TRAP, and eAls proteins with 0.1 mM isopropyl‐β‐D‐1‐thiogalactopyranoside (IPTG, Biosharp) induction at 37°C for 4 h, respectively. Then, the bacterial cells were obtained by centrifugation and were ultrasonicated, and the suspension was acquired. The His‐tagged ATT, TRAP, and eAls proteins were purified by using His‐binding‐resin (Novagen) according to the manufacturer's instructions. These proteins were confirmed with sodium dodecyl sulphate–polyacrylamide gel electrophoresis (SDS‐PAGE) and Western blot. For Western blot, anti‐His tag monoclonal antibodies (mAbs) (Sigma) and horseradish peroxidase (HRP)‐conjugated goat antimouse IgG antibodies (Sigma) were used as the primary antibodies, the secondary antibodies, respectively.

### 2.4 Mice immunization

After ATT proteins were prepared, we next assessed their immunogenicity. Hundred C57/B6 mice were randomly divided into five groups, including ATT, TRAP, eAls, TRAP + eAls, and phosphate buffer solution (PBS) group, there were 20 mice in each group. C57/B6 mice were immunized intramuscularly at the dosage of the solution with ATT, TRAP, eAls, TRAP + eAls of 100 μg or PBS emulsified with equal volume Freund's incomplete or complete adjuvant (Sigma‐Aldrich) to a final volume of 0.2 ml on 0 day and 21 days, respectively. All the animals were fed in a special pathogen‐free environment.

In addition, 160 C57/B6 mice were randomly divided into eight groups, including ATT + CpG + MDP + FIA, ATT + MDP + FIA, ATT + CpG + FIA, ATT + CpG + MDP, ATT + CpG, ATT + MDP, ATT + FIA, PBS + FIA group. Briefly, CpG (the sequence: (5′‐TCCATGACGTTCCTGACGTT‐3′)) was synthesized by gene synthesis corporation (Sangon Biotech Co., Ltd). The dosage of ATT, CpG, and MDP (Sigma‐Aldrich, A9519) was 100 μg, 10 ng, and 10 ng, respectively. ATT solution was emulsified with FIA at a volume ratio of 1:1, and ATT solution plus CpG and MDP or alone CpG or MDP was emulsified with FIA at a volume ratio of 1:1. FIA was emulsified with PBS at a volume ratio of 1:1. Each mouse was immunized intramuscularly at a dose of 200 μl in the muscle of the lateral thigh. The booster immunization was performed on 21 days after the primary immunization.

### 2.5 Detection of cytokine

The amounts of cytokine was detected using enzyme‐linked immunospot (ELISpot) assay or enzyme‐linked immuno sorbent assay (ELISA), respectively. ELISpot was performed according to the kit instructions. Briefly, lymphocytes were separated from spleens in mice with lymphocyte separation fluid, then, spleen lymphocytes (1 × 10 ^6^ cells/well) were seeded into 96‐well culture plates and cultured in 1640 medium supplemented with 10% foetal bovine serum at 37°C for 24 h, either alone as negative control or with phorbol myristate acetate (PMA, 50 ng/ml) as positive control or with the desired proteins (TRAP, 10 µg/ml). The data were expressed as the number of spot‐forming cells (SFCs)/10 ^6^ lymphocytes. For cytokine profile analysis, the treated cells were cultured at 37°C for 48 h, then, the supernatant were collected and analyzed with ELISA.

### 2.6 Analysis of specific antibodies

The sera were separated from blood samples in mice 2 weeks after booster immunization. IgG antibodies against TRAP in sera were detected by ELISA as described previously.^37^ Briefly, TRAP proteins were used to coat the 96‐well plates at concentration of 10 μg/ml and incubated at 4°C overnight. After washing three times with phosphate buffer solution tween‐20 (PBST), the plates were blocked with 3% bovine serum albumin at 4°C for 2 h, then, the samples of twofold serial dilution were added into the wells and incubated at room temperature (RT) for 2 h. After washing with PBST, HRP‐conjugated goat antimouse IgG or IgG1, IgG2a, IgG2b, IgG3 mAbs were added and incubated at RT for 1 h. After washing, the plates were incubated with 3, 3′, 5, 5′‐tetramethylbenzidine (TMB) solution for 15 min, then, 2 N sulfuric acid was added into the plates. Finally, OD_450nm_ (Optical Densities) value was measured with an automated ELISA plate reader.

### 2.7 Challenge assay

To assess the immune‐protective effect, the challenge assay was performed. Two weeks after booster immunization, the C57/B6 mice from the challenge groups were infected intraperitoneally with the lethal dose of 8 × 10^8^ colony‐forming units (CFU) of *S. aureus* strain Newman and 6 × 10^8^ CFU of *S. aureus* strain Wood46, respectively. The mice were detected for mortality and recorded every day after infection. Finally, the survival rate of mice from each group was determined after 14 days.

### 2.8 Statistical analysis

All data were analyzed by using the SPSS13.0 statistical software. The data were presented as the means plus standard deviations. Differences groups were identified by Student's *t* test. Differences were considered significant at *p* < .05 or *p* < .01 and *p* < .001.

## 3 RESULTS

### 3.1 Confirmation of ATT, TRAP, and eAls expression

**Figure 2:**
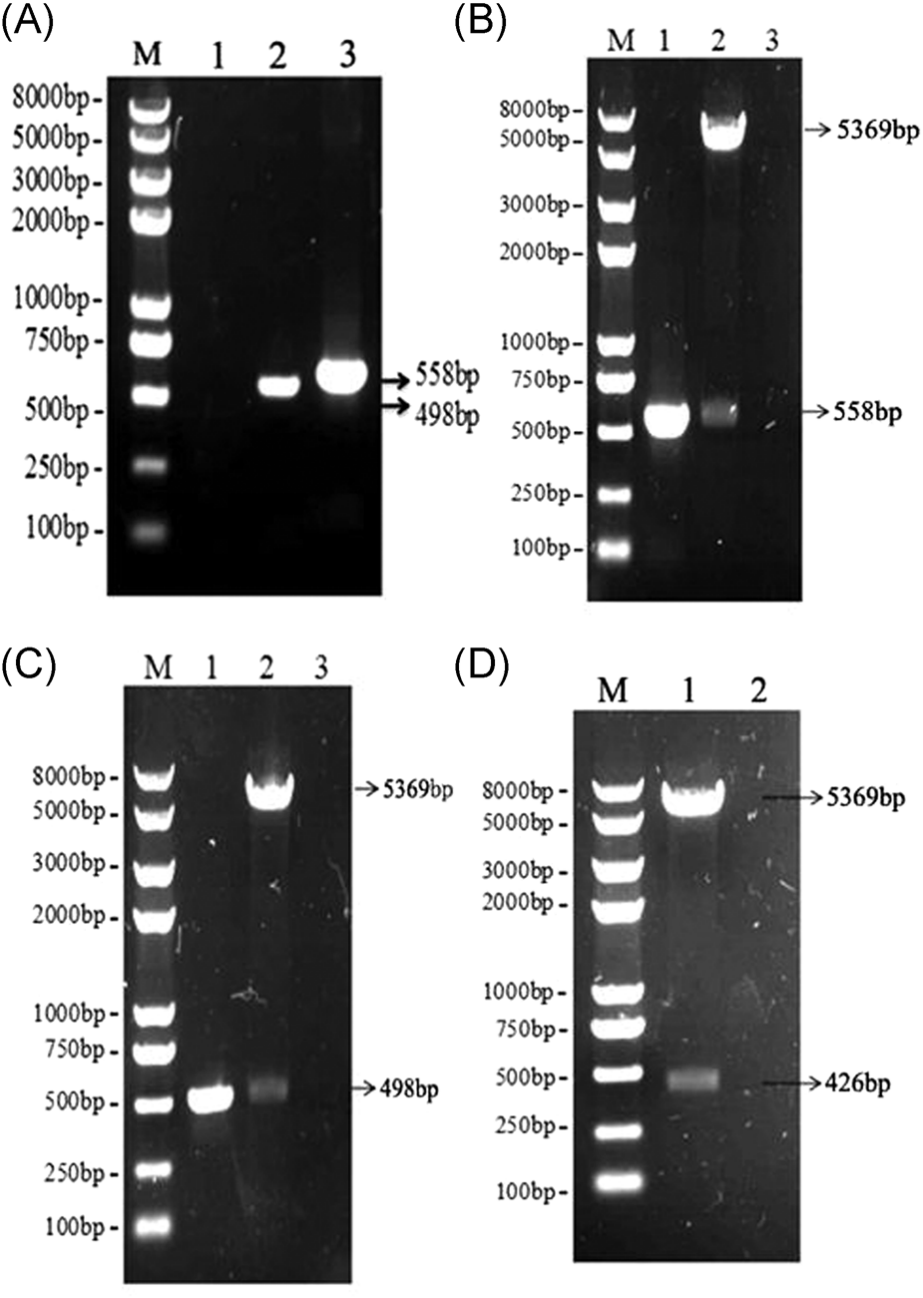
Acquisition of target fragments and construction of recombinant plasmids. (A) Acquisition of target fragments. The fragments of 498 and 558 bp were obtained using PCR method, respectively. M lane: DNA Marker; 1 lane: Mock; 2 lane: PCR products of 498 bp; 3 lane: PCR products of 558 bp. (B) The identification of the pET‐28a(+)‐*att*plasmids. The analytical results showed the fragments of 558 bp were obtained with PCR, the digestion of restriction endonucleases, respectively. M lane: DNA Marker; 1 lane: PCR products; 2 lane: Digestion products; 3 lane: Mock. (C) The identification of the pET28a‐(+)‐*trap*plasmids. The fragments of 498 bp are exhibited with PCR and the digestion of restriction endonucleases, respectively. M lane: DNA Marker; 1 lane: PCR products; 2 lane: Digestion products; 3 lane: Mock. (D) The confirmation of the pET‐28a‐(+)‐*eAls*plasmids. The segments of 426 bp are displayed with the digestion of restriction endonucleases. M lane: DNA Marker; 1 lane: Digestion products; 2 lane: Mock. PCR, polymerase chain reaction

To obtain recombinant pET‐28a (+)‐*att* plasmids, the *att* fragments were amplified from pET‐32a (+)‐*trap* with a pair of specific primers carrying the nucleotide sequence of *Als3 epitope*, and the nucleotide sequence encoding a GSGSGSGS linker was introduced between *Als3 epitope* and *trap* fragment to maintain the native conformation of Als3‐TRAP protein. The segments of 558 bp were obtained with PCR (Figure 2A) and linked into pET‐28a (+) plasmids, and the results showed the fragments of 558 bp were exhibited in Figure 2B with PCR and digestion method, indicating the recombinant pET‐28a (+)‐*att* plasmids were correctly constructed. The segments of 498 bp were acquired using PCR (Figure 2A), and after pET‐28a (+)‐ *trap* plasmids were treated with *Bam*H I/*Hin*d III restriction endonucleases and PCR method, the segments of 498 bp were displayed in Figure 2C. The segments of 426 bp were obtained after pET‐28a (+)‐*eAls* plasmids were digested with the double restriction endonucleases (Figure 2D). In addition, all of the above plasmids were verified by sequencing analysis. Therefore, these data demonstrated that the desired recombinant plasmids were correctly constructed.

**Figure 3:**
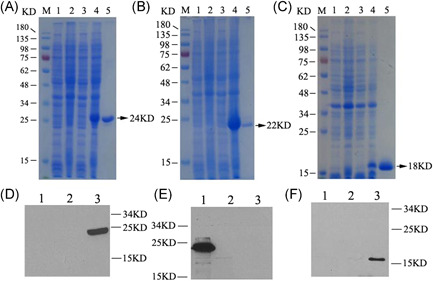
Analysis of protein expression. 1. (A), (B), (C) The expression of target proteins. The desired proteins, ATT (A), TRAP (B) and eAls (C) are exhibited with the molecular weight of 24, 22, and 18 KD with SDS‐PAGE assay, respectively. (A), (B), (C): M lane: Protein Marker; 1 lane: The noninduced recombinant bacteria with pET‐28a (+) plasmids; 2 lane: The induced recombinant bacteria with pET‐28a (+) plasmids. (a): 3 lane: The noninduced recombinant bacteria with pET‐28a (+)‐*att* plasmids. 4 lane: The induced recombinant bacteria with pET‐28a (+)‐*att* plasmids. 5 lane: The purified ATT proteins. (b): 3 lane: The noninduced recombinant bacteria with pET‐28a (+)‐*trap* plasmids. 4 lane: The induced recombinant bacteria with pET‐28a (+)‐*trap* plasmids. 5 lane: The purified TRAP proteins. (C): 3 lane: The noninduced recombinant bacteria with pET‐28a (+)‐*eAls* plasmids. 4 lane: The induced recombinant bacteria with pET‐28a (+)‐*eAls* plasmids. 5 lane: The purified eAls proteins. 2. (D), (E), (F) Identification of the desired proteins. The expression of ATT (D), TRAP (E), and eAls (F) proteins was confirmed with Western blot. (D): 1 lane: The induced *E. coli* strain BL21; 2 lane: The induced *E. coli* strain BL21 with pET‐28a (+) plasmids; 3 lane: The induced *E. coli* strain BL21 with pET‐28a (+) –*att* plasmids. (E): 1 lane: The induced *E. coli* strain BL21 with pET‐28a (+)–*trap* plasmids; 2 lane: The induced *E. coli* strain BL21 with pET‐28a (+) plasmids; 3 lane: The induced *E. coli* strain BL21. (F): 1 lane: The induced *E. coli* strain BL21; 2 lane: The induced *E. coli* strain BL21 with pET‐28a (+) plasmids; 3 lane: The induced *E. coli* strain BL21 with pET‐28a (+) –*eAls* plasmids. SDS‐PAGE, sodium dodecyl sulphate–polyacrylamide gel electrophoresis; TRAP, Target of RNAIII Activating Protein

SDS‐PAGE results indicated that ATT (24 kDa) (Figure 3A), TRAP (22 kDa) (Figure 3B), eAls (18 kDa) (Figure 3C) proteins were expressed by the recombinant *E. coli* BL21 (DE3) strains with pET‐28a (+)‐*att*, pET‐28a (+)*‐trap*, and pET‐28a (+)‐*eAls* plasmids, respectively, and these proteins were successfully purified (Figure 3A‐C). Western blot results confirmed that the size of bands was consistent with the expected molecular weight of the desired proteins, ATT (Figure 3D), TRAP (Figure 3E), and eAls (Figure 3F), respectively, indicating these proteins were successfully expressed.

### 3.2 Als3 epitopes enhanced TRAP immunogenicity

#### 3.2.1 Als3 epitopes increased cytokine production from lymphocytes

**Figure 4:**
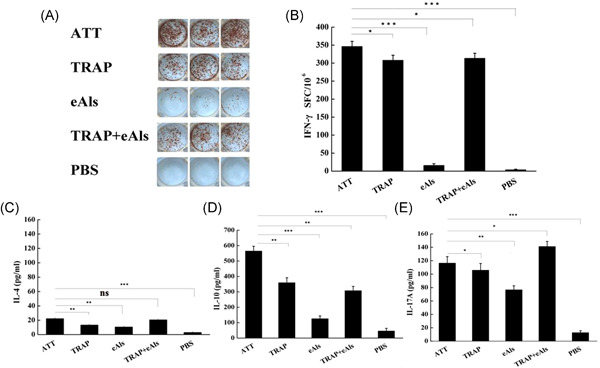
Evaluation of cytokine production. (A) The scanned images of ELISpot membranes. Representative images of spot forming cells (SFC) for IFN‐γ secretion are exhibited. (B) ELISpot analysis. Data represent the mean of spot‐forming cells (SFCs) per million splenocytes with the standard deviation (SD). (C), (D), (E) The measurement of ELISA. IL‐4 (C), IL‐10 (D) and IL‐17A (E) cytokines from cell supernatants were detected by ELISA. Data are shown as means ± *SD* (*n* = 3). Statistical differences between groups are shown as *p < .05 or **p < .01 and *** *p* < .001, and the differences were not considered significant at *p* > .05 (ns). ELISpot, enzyme‐linked immunospot

To assess the cellular immune responses elicited with the desired antigens, the IFN‐γ level from spleen lymphocytes in each group was determined with ELISpot. The representative images of TRAP‐specific spot forming cells are shown in Figure 4A. The results showed that IFN‐γ level from ATT group was higher than TRAP, TRAP + eAls group, and IFN‐γ level from ATT group was significantly different from that eAls group (Figure 4A,B). In addition, ELISA assay was performed to detect the level of IL‐4, IL‐10, and IL‐17A in the supernatant of spleen lymphocytes. The results indicated the level of IL‐4 and IL‐10 in ATT group was significantly different from those in TRAP and eAls group, and the level of IL‐10 from ATT group was significantly different from those in TRAP + eAls group (*p* < .01), but the level of IL‐4 in ATT group was not significantly different from those in TRAP + eAls group (*p* > .05) (Figure 4C,D), in addition, the level of IL‐17A in ATT group was higher than TRAP group and was significantly different from that of eAls group (*p* < .01), but slightly lower than TRAP + eAls group (Figure 4E).

#### 3.2.2 Als3 epitopes enhanced humoral immune response

**Figure 5:**
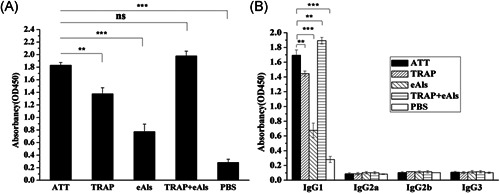
Detection of antibodies in serum. (A), (B) The level of IgG antibodies and IgG subclasses (IgG1, IgG2a, IgG2b, and IgG3) against TRAP proteins are showed in figure (A) and (B), respectively. The level of IgG antibodies against TRAP was assessed with ELISA. The OD_450nm_ value is shown as means ± *SD* (*n* = 3), and the significant differences were expressed as **p < .01 or ***p < .001, and the differences were not considered significant at *p* > .05 (ns)

ELISA was performed to evaluate the level of antibodies against TRAP in sera from mice. As shown in Figure 5A, the level of IgG antibodies against TRAP from TAA group was higher than TRAP and eAls groups, but slightly low compared with TRAP + eAls group (Figure 5A). In addition, ELISA was used to detect the level of IgG subclasses in each group. As shown in Figure 5B, the results displayed IgG1 level was the highest among IgG1, IgG2a, IgG2b, and IgG3 subclasses, and the level of IgG1 from ATT group was higher than from TRAP and eAls groups, but low compared with TRAP + eAls group (Figure 5B).

#### 3.2.3 Als3 epitopes boosted protective immune response

**Figure 6:**
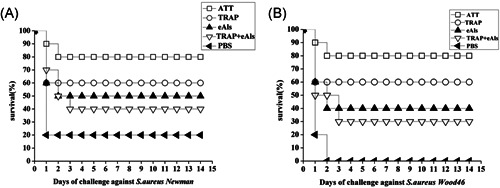
Survival rate of mice after challenge. (A), (B) Assessment of immune protection response. Two weeks after the boost immunization, the immunized mice were challenged with the lethal dose of 8 × 10^8^ CFU of *S. aureus* strain Newman (A) and 6 × 10^8^ CFU of *S. aureus* strain Wood46 (B), respectively, then, the survival of mice was recorded for 14 days after *S. aureus* infection. CFU, colony‐forming units

The immunized mice were challenged with *S. aureus* Newman strain and *S. aureus* Wood46 strain, respectively. After 3 days challenged with *S. aureus* Newman strain, the results showed the immune survival rate of 80% in ATT group was the highest among all groups, but PBS group exhibited the lowest immune survival rate of 20%, 60%, 50%, and 40% was in TRAP, eAls and TRAP + eAls groups, respectively (Figure 6A). After the mice were infected with *S. aureus* Wood 46 strain for 3 days, all of the mice in PBS group died, and the highest immune survival rate was 80% in ATT group, 60% was in TRAP group, 40% was in eAls group, and 30% was in TRAP + eAls group (Figure 6B). These data indicated that Als3 epitopes obviously boosted the protective immune response of TRAP.

### 3.3 The combined adjuvants improved cytokine production from lymphocytes

**Figure 7:**
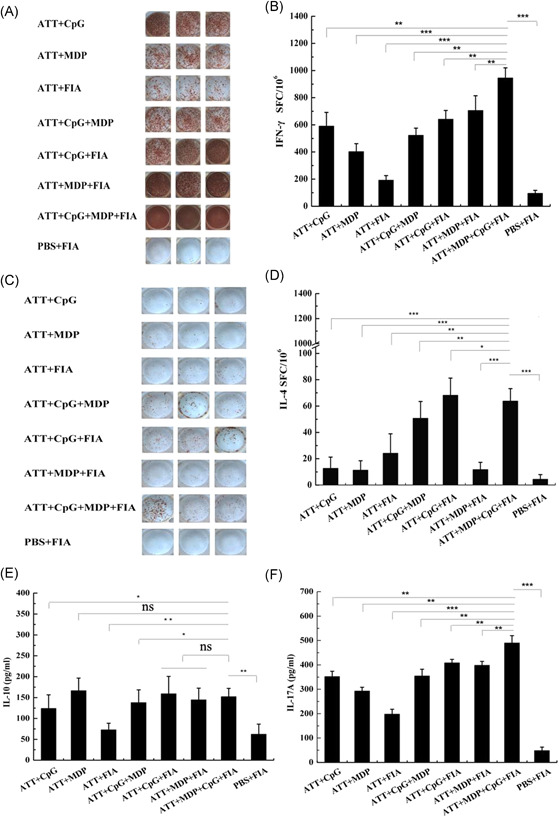
Analysis of cytokine secretion. (A), (C) The representative scanned images of ELISpot. IFN‐γ and IL‐4 secretion of splenocytes from each group was determined with ELISpot assay. The representative scanned images about IFN‐γ (a) and IL‐4 (c) secretion are exhibited, respectively. (B), (D) Analysis of ELISpot data. Data represent the mean of TRAP‐specific spot‐forming cells (SFCs) for IFN‐γ (b) and IL‐4 (d) secretion per million splenocytes. Data are shown as means ±* SD* (*n* = 3). (E), (F) The detection of IL‐10 and IL‐17A. The level of IL‐10 (E) and IL‐17A (F) in the supernatants of splenocytes was detected with ELISA. Data are shown as means ± *SD* (*n* = 3). Statistical differences between groups are shown as *p < .05 or **p < .01 and ***p < .001, and the differences were not considered significant at *p* > .05 (ns). ELISA, enzyme‐linked immuno sorbent assay; ELISpot, enzyme‐linked immunospot; IFN‐γ, interferon‐γ; IL‐4, interleukin‐4

The secretion of IFN‐γ and IL‐4 from spleen lymphocytes was determined by ELISpot. The representative images of TRAP‐specific spot forming cells for IFN‐γ secretion were shown in Figure 7A, the statistic results showed the production of IFN‐γ in ATT + CpG + MDP + FIA group was highest in all groups (Figure 7B). Furthermore, the representative images of TRAP‐specific spot forming cells for IL‐4 secretion were exhibited in Figure 7C, the statistic results manifested the secretion of IL‐4 in ATT + CpG + MDP + FIA group was significantly higher than other control groups, but was slightly low compared with ATT + CpG + FIA group (Figure 7D).

IL‐10 and IL‐17A level from spleen lymphocytes in each group was analyzed by ELISA assay. As shown in Figure 7E, IL‐10 level in ATT + CpG + MDP + FIA group was significantly higher PBS + FIA, ATT + CpG, ATT + FIA and ATT + CpG + MDP groups, but significant difference was not exhibited in comparation with other control groups (Figure 7E). In addition, the results showed that IL‐17A level in ATT + CpG + MDP + FIA group was significantly higher than control groups (Figure 7F), indicating the combination of CpG + MDP + FIA markedly promoted IL‐17A production. In general, IL‐4 and IL‐10 are characteristic of Th2‐type immune response and activate B cells. Th1‐type immune response is characterized by secreting IFN‐γ. IL‐17 from Th17 cells promotes neutrophils recruitment to elicit inflammation. From these above results, the combined adjuvants of CpG, MDP, and FIA are able to induce Th1, Th2, and Th17 cell activating, as a result, the T‐cell‐mediated immune responses are obviously strengthened.

### 3.4 The combined adjuvants increased the level of antibodies

**Figure 8:**
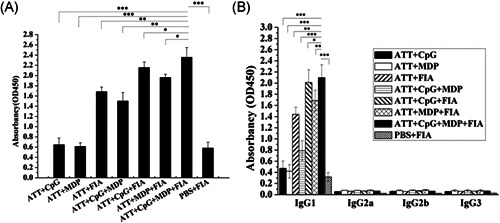
Detection of humoral immune response. (A), (B) Evaluation of antibodies in serum from mice immunized with ATT plus the corresponding adjuvant. Two weeks after the boost immunization, the level of IgG (A) and IgG subclasses (B) (IgG1, IgG2a, IgG2b, and IgG3) against TRAP in serum from immunized mice was detected by ELISA. The data are shown as the mean ± *SD* (*n* = 3). Statistical differences between groups are shown as *p < .05 or **p < .01 and ***p < .001. ELISA, enzyme‐linked immuno sorbent assay; TRAP, Target of RNAIII Activating Protein

The level of IgG antibodies against TRAP in the sera from mice was detect with ELISA. As shown in Figure 8A, the sera from ATT + CpG + MDP + FIA group exhibited the highest IgG level among all groups, and obviously higher than PBS + FIA, ATT + CpG, ATT + MDP, ATT + FIA, ATT + CpG + MDP groups (Figure 8A). Furthermore, the analysis of IgG subclasses showed that IgG1 level was the highest level among IgG1, IgG2a, IgG2b, and IgG3 subclasses, and the sera from ATT + CpG + MDP + FIA was the highest IgG1 level among all groups, and obviously higher than PBS + FIA, ATT + CpG, ATT + MDP, ATT + FIA, ATT + CpG + MDP groups (Figure 8B). These results showed that Als3 epitopes plus CpG, MDP and FIA adjuvant significantly increased the humoral immune response triggered with TRAP.

### 3.5 The combined adjuvants strengthened protective immune response

**Figure 9:**
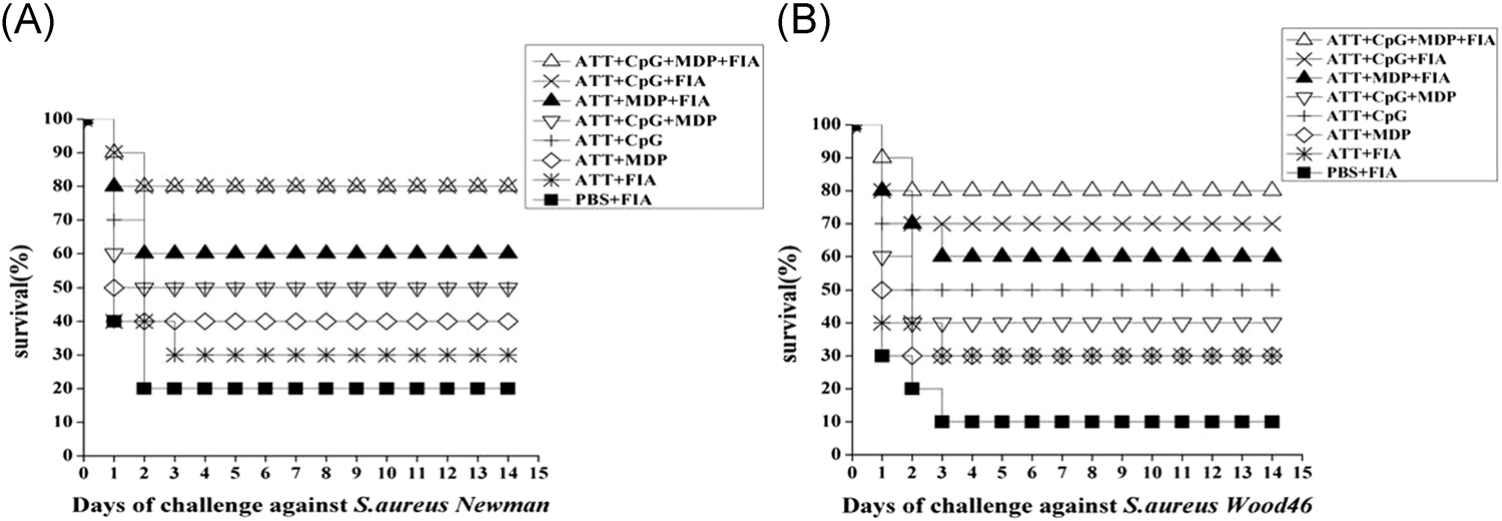
Determination of immune protection in mice. (A), (B) Measurement of the immune protection triggered with ATT plus the corresponding adjuvant in mice. The immunized mice were challenged with the lethal doses of *S. aureus* strain Newman (A) and *S. aureus* strain Wood46 (B), respectively. Then, the mice were detected for mortality and recorded every day after infection. The survival of mice was determined 14 days after challenge

Two weeks after the boost immunization, challenge assay was performed by using *S. aureus* strain Newman and *S. aureus* Wood46 strain, respectively. As shown in Figure 9A, after 3 days challenged with *S. aureus* Newman strain, the results indicated the lowest survival rate in PBS + FIA group was 20%, and 80% in ATT + CpG + MDP + FIA and ATT + CpG + FIA groups was the highest among all groups, but 60%, 50%, 50%, 40%, and 30% was in ATT + MDP + FIA, ATT + CpG + MDP, ATT + CpG, ATT + MDP, ATT + FIA groups, respectively (Figure 9A). After the mice were infected with *S. aureus* Wood 46 strain for 3 days, the immune survival rate of 10% displayed in PBS + FIA group, and the highest immune survival rate of 80% was in ATT + CpG + MDP + FIA group, 70% was in ATT + CpG + FIA group, 60% was in ATT + MDP + FIA group, 50% and 40% was exhibited in ATT + CpG and ATT + CpG + MDP groups, respectively, 30% was in ATT + MDP and ATT + FIA groups (Figure 9B). These data indicated that Als3 epitopes plus the combined adjuvants obviously enhanced the protective immune response triggered with TRAP.

## 4 DISCUSSION

**Figure 10:**
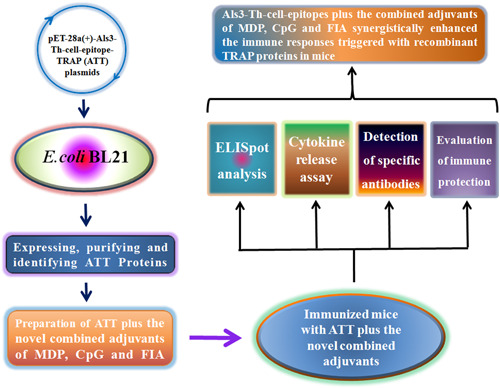
The graphical table of contents. The recombinant ATT proteins were expressed and prepared. ATT plus the novel combined adjuvants of MDP, CpG, and FIA induced the strong immune response and protection against *S. aureus* in mice, revealing the novel combined adjuvant acts the synergistic effect.

In this study, ATT proteins were prepared by fusion expression of *Als*3 epitope and *trap*, and Als3 epitopes were able to increase obviously TRAP immunogenicity. Additionally, Als3 epitopes plus the combined adjuvants of CpG, MDP, and FIA strengthened the immune response of TRAP (Figure 10). These data would provide a novel strategy for enhancing the immune protection of vaccines against *S. aureus*.

Recently, TRAP proteins have exhibited the great potential as a new vaccine candidate. TRAP proteins, as immunogens, can effectively inhibit the generation of *S. aureus* toxins, and display the protective effect against *S. aureus* infection.^38^ However, the TRAP immunogenicity is not up to the desired requirements, therefore, this study was performed to further enhance TRAP immunogenicity.

Owing to Als3 similar to three‐dimensional structures of *S. aureus* ClfA, and Als3 epitopes to trigger across‐immune response against *S. aureus* and *C. albicans* infection,^23^, ^24^, ^25^ we prepared ATT proteins to confirm if Als3 epitopes promote TRAP immunogenicity. First, the level of IL‐4, IL‐10, IL‐17, and IFN‐γ from ATT group were significantly higher than TRAP group. Second, the level of IgG antibodies against TRAP in ATT group was higher than TRAP group, moreover, IgG1 generation was significantly different between ATT group and TRAP group, indicating that ATT proteins are able to elicit the strong humoral immune response in mice. Finally, the challenged results showed the immune protection of ATT was obviously stronger than TRAP, indicating that Als3 epitopes were able to enhance the immune protective effect of TRAP. Therefore, our data demonstrated that Als3 epitopes markedly enhanced the TRAP immunogenicity by their fusion expression.

CpG and MDP can improve the development of cellular immune response toward Th1/Th17 cells.^39^, ^40^ FIA can adsorb antigens and activate innate immunity, moreover, it can slow antigen to release into the microenvironment and extend antigen‐induced immune responses in vivo. Recent studies reveal that the combined adjuvants acts the synergic effect and obviously enhances the protective immune responses of antigens.^41^ Therefore, the combined adjuvants are new trends in the vaccine development. Presently, coactions of different adjuvants have been gradually utilized, for example, the combined utilization of CpG ODN and nanoemulsion adjuvant (NE02), CpG ODN and Poly(I:C) (Polyinosinic‐polycytidylic acid), streptavidin‐4‐1BBL (SA‐4‐1BBL) and monophosphoryl lipid A (MPLA), aluminum salts and CpG ODN plus innate defense regulator peptide HH2, which has attained the desired immune response of antigens.^42^, ^43^, ^44^, ^45^ In recent years, IL‐17 and IFN‐γ play a crucial role for killing *S. aureus*. IL‐17 can recruit neutrophils to eliminate *S. aureus*, IFN‐γ activates macrophages to engulf and kill *S. aureus*.^46^ IL‐17 and IFN‐γ were mainly secreted from Th1 cells, Th17 cells, respectively. Thus it could be indicated that Th1/Th17‐mediated immune response plays a key role in killing *S. aureus*. Hence, on the development of vaccines against *S. aureus* infection, it is particularly important for alliance of different adjuvants to active Th1 and Th17 cells. Therefore, in this study, we prepared the combine adjuvants of CPG, MDP, and FIA to enlarge the immune protective effect of ATT against *S. aureus* infection.

Cytokine detection revealed the secretion levels of IL‐17 and IFN‐γ from ATT + CpG + MDP + FIA group were significantly higher than control groups, which indicated that Th1 and Th17 cells were activated. However, it will be further performed to evaluate Th1/Th17 cell‐mediated immune response triggered with ATT plus CpG, MDP and FIA adjuvant in future. By detecting the humoral immune response, it was found that the level of IgG antibodies against TRAP in ATT + CpG + MDP + FIA group was not significant difference than ATT + CpG + FIA and ATT + MDP + FIA groups, but there was significant difference compared with other experimental groups. In addition, IgG1 level in ATT + CpG + MDP + FIA group was the highest in all groups, indicating that ATT plus CpG, MDP and FIA adjuvant generated the strong humoral immune response. Finally, in the challenge experiment, the immune results shown that ATT + CpG + MDP + FIA group exhibited 80% survival rate against *S. aureus* strain Newman and *S. aureus* strain Wood46, which was obviously higher than the control groups. Therefore, the Als3 epitopes plus the combined adjuvants of CpG, MDP, and FIA were able to generate the synergistic effect to enhance the immune response of TRAP. However, to further increase TRAP immunogenicity, other TLR agonists, for example, Poly (I:C), MPLA and so on, will be considered in conjugation with ATT in future research.^47^, ^48^, ^49^ In this study, we only selected *S. aureus* strain Newman and *S. aureus* strain Wood46 as the challenge strains, and other *S. aureus* strains will be used to further evaluate the immune protection triggered with ATT plus the combined adjuvants of CpG, MDP and FIA in the future.

## 5 CONCLUSIONS

Taken together, our data showed that ATT proteins successfully obtained, and Als3 epitopes significantly strengthened the immune response of TRAP, the novel combined adjuvants of CpG, MDP, and FIA exhibited the strong synergy, and significantly heightened TRAP‐induced immune responses in conjunction with Als3 epitopes (Figure 10), which will provide an important basis for the development of vaccines against *S. aureus* infection.

## CONFLICT OF INTERESTS

The authors declare that there are no conflict of interests.

## AUTHOR CONTRIBUTIONS

JinZhu Ma contributed design of the study and analyzed the data. Wei Liu performed the experiments. Beiyan Wang, Simiao Yu, Liquan Yu, Baifen Song, Yongzhong Yu, and Zhanbo Zhu contributed to data collection.

## Data Availability

The data that support the findings of this study are available from the corresponding author upon reasonable request.
